# Calorie Restriction Improves Physical Performance and Modulates the Antioxidant and Inflammatory Responses to Acute Exercise

**DOI:** 10.3390/nu12040930

**Published:** 2020-03-27

**Authors:** Xavier Capó, Miquel Martorell, Miguel D. Ferrer, Antoni Sureda, Victoria Pons, Juan C. Domingo, Franchek Drobnic, Alejandro Martínez-Rodríguez, Belén Leyva-Vela, José M. Sarabia, María Herranz-López, Enrique Roche, Josep A. Tur, Antoni Pons

**Affiliations:** 1 Laboratory of Physical Activity Science, Research Group on Community Nutrition and Oxidative Stress, University of Balearic Islands, 07122 Palma de Mallorca, Balearic Islands, Spain; xavier.capo@uib.es (X.C.); mmartorell@udec.cl (M.M.); miguel-david.ferrer@uib.es (M.D.F.); tosugo@hotmail.com (A.S.); pep.tur@uib.es (J.A.T.); 2 IDISBA. Fundació Institut d’Investigació Sanitària Illes Balears, Hospital Universitari Son Espases, 07120 Palma de Mallorca, Balearic Islands, Spain; 3 Nutrition and Dietetics Department, Faculty of Pharmacy, University of Concepcion, 4070386 Concepcion, VIII – Bio Bio Region, Chile; 4 CIBEROBN (Physiopathology of Obesity and Nutrition), Instituto de Salud Carlos III (CB12/03/30038), 28220 Madrid, Spain; eroche@umh.es; 5 Performance and Health Research Group for High Level Sports, High Performance Center of Barcelona, 08174 Sant Cugat, Spain; vpons@car.edu (V.P.); drobnic@car.edu (F.D.); 6 Department of Biochemistry and Molecular Biology, University of Barcelona, 08028 Barcelona, Spain; jcdomingo@ub.edu; 7 Department of Analytical Chemistry, Nutrition and Food Science, Alicante University,03690 Alicante, Spain; amartinezrodriguez@ua.es; 8 Vinalopó Hospital, 03293 Elche, Spain; bmleyva@vinaloposalud.com; 9 Sport Research Center, University Miguel Hernández, 03202 Elche, Spain; jsarabia@goumh.umh.es; 10 Institute of Research, Development, and Innovation in Biotechnolgy of Elche (IDiBE) and Molecular and Cell Biology Institute (IBMC), University Miguel Hernández, 03202 Elche, Spain; mherranz@umh.es; 11 Institute of Bioengineering and Department of Applied Biology-Nutrition, University Miguel Hernández. Alicante Institute for Health and Biomedical Research (ISABIAL Foundation), 03010 Alicante, Spain

**Keywords:** calorie restriction, exercise, oxidative stress, fatty acids, plasma

## Abstract

Our aim was to characterize the effects of calorie restriction on the anthropometric characteristics and physical performance of sportsmen and to evaluate the effects of calorie restriction and acute exercise on mitochondria energetics, oxidative stress, and inflammation. Twenty volunteer taekwondo practitioners undertook a calorie restriction of 30–40% on three alternate days a week for one month. Eleven volunteer sportsmen participated as controls. Both groups performed an energy efficiency test to evaluate physical performance, and samples were taken before and after exercise. The total weight of participants significantly decreased (5.9%) after calorie restriction, while the efficiency of work and the contributions of fat to obtain energy were enhanced by calorie restriction. No significant differences induced by acute exercise were observed in individual non-esterified fatty acid percentage or oxidative stress markers. Calorie restriction downregulated the basal gene expression of nitric oxide synthase, antioxidant enzymes, mitochondrial uncoupling proteins, and repairing stress proteins, but it enhanced the expression of sirtuins in peripheral blood mononuclear cells. In conclusion, one month of calorie restriction decreases body weight and increases physical performance, enhancing energy efficiency, moderating the antioxidant and inflammatory basal gene expression, and influencing its response to acute exercise.

## 1. Introduction

Calorie restriction is a dietary strategy usually based on decreasing the calorie intake (about 20–40% of the ad libitum diet) without challenging the intake of essential nutrients [1]. This intervention prolongs the lifespan of rats [2] and other organisms [3], including higher mammals such as rhesus monkeys [4,5]. Moreover, studies in rodents show that calorie restriction attenuates atherosclerosis, obesity, and diabetes-related vascular dysfunction [6,7,8], while studies in humans and monkeys show beneficial effects on diseases such as type 2 diabetes, obesity, inflammation, hypertension, cancer, and cardiovascular diseases [3,9,10,11]. Different studies have provided mechanistic perspectives on the effects of calorie restriction on vascular homeostasis, including the attenuation of oxidative stress and inflammation and the enhancement of nitric oxide bioactivity [12,13,14,15]. Calorie restriction mediates on vasculature through a number of target molecules such as sirtuins, AMP-activated protein kinase, mammalian targets of rapamycin, and endothelial nitric oxide synthase, as well as their regulatory pathways [12]. Calorie restriction induces the proliferation of mitochondria with more efficient electron transport systems, which is related to decreased whole-body oxygen utilization and attenuation of oxidative damage in mitochondrial DNA and other cellular organelles [16].

In humans, calorie restriction is also associated with decreased oxidative stress and inflammatory markers [13,15]. At the cellular level, one of the hallmark features of calorie restriction is the improvement of mitochondrial metabolism and function, which ultimately reduces oxidative damage [17]. Calorie restriction induces the upregulation of genes involved in mitochondrial function and biogenesis, such as peroxisome proliferator-activated receptor gamma co-activator 1 alpha (PGC1α), mitochondrial transcription factor A (TFAM), silent information regulator (SIRT1), and endothelial nitric oxide synthase (eNOS) in the skeletal muscle [16]. The combination of calorie restriction with exercise increases aerobic fitness in parallel with improved insulin sensitivity, decreasing LDL-cholesterol, and diastolic blood pressure [18]. Three weeks of calorie restriction in normal-weight men reduced leucine flux and oxidation during exercise with a negative nitrogen balance, induced loss of lean mass, and maintained whole-body exercise performance [19].

Calorie restriction strategies include fasting periods in which fatty acids are mobilized from intracellular stores to provide energy to working muscles. In this context, the fatty acid composition of the diet is known to influence the fatty acid composition of plasma/serum lipids [20,21,22,23]. Regarding this potential for fatty acid mobilization, calorie restrictions and exercise are instrumental strategies to induce significant weight loss. Nevertheless, the combination of both strategies together has not been properly addressed [24,25,26]. Therefore, the aim of this study was to evaluate the effect of calorie restriction, compared to a free diet, on physical performance and body composition in a group of taekwondo practitioners. In addition, the combined effects of calorie restriction and acute exercise were assessed on oxidative damage, the expression of genes involved in the regulation of mitochondrial function, biogenesis and inflammation, and on the availability of non-esterified fatty acids (NEFAs).

## 2. Materials and Methods

### 2.1. Participants and Study Design

Thirty-one well-trained male taekwondo practitioners agreed to participate voluntarily in this study. The inclusion criteria were age (18–50 years old), gender (male), non-smokers, and performing physical activity 3 days a week. All athletes who participated in the study, were professional athletes from the High-Performance Center of Barcelona (Barcelona, Spain). All participants followed a very strict training program, training between 3 and 5 days per week for 2–3 hours. Training seasons included workout on fitness, technique, and combat. This sport was chosen because athletes often have to lose weight acutely and are more likely to follow such demanding calorie restrictions. Most of participants competed at the national and international level. All the subjects were informed regarding the objectives, requirements, and possible risks of the study before giving their written consent. Before being accepted to participate in the research, each subject passed a complete medical examination, which included a medical history and resting electrocardiogram (ECG). The study protocol was in accordance with the Declaration of Helsinki for research on human beings and was approved by the local Ethics Committee of the Consell Català de l’Esport (Catalan Sports Council; Ref. 07033C0301). The project was registered at ClinicalTrials.gov (NCT02533479).

Participants were distributed between the control group and the calorie restriction group. The control group included 11 healthy sportsmen who followed a complete diet during the entire study. The calorie restriction group was formed by 20 healthy sportsmen who followed a diet with 30–40% calorie restriction for 1 month (4 weeks). The macronutrient composition of both diets was similar at the beginning of the calorie restriction period (Table 1). Control and calorie restriction participants executed two exercise energy efficiency tests to evaluate physical performance: the first at the beginning and the second at the end of the study. Blood samples were obtained before and after the physical performance tests.

All of the participants were informed of the purpose and demands of the study before providing their written consent to participate. The protocol complied with the Declaration of Helsinki for research on human subjects and was approved by the Clinical Research Ethics Committee at the Direcció General de l’Esport of the Catalonian Sports Council (Ref. 07033C0301).

### 2.2. Calorie Restriction Prescription

Each participant was interviewed regarding their dietary, lifestyle, and training habits. The nutritional value of their daily diet was assessed using a 7-day food record, including all foods and beverages consumed, specifying the servings, cooking techniques, and daily distribution. From this information, a diet analysis was performed using a computer program based on food composition tables, CESNID [27].

Over a period of six weeks, the participants practiced an every-other-day fasting calorie restriction (CR) program, decreasing calorie intake by 33% with respect to their usual diets. The participants restricted their habitual diets for three alternate days each week and on the other four days participants’ dietary intakes were the same as they were at the beginning of the study without changes to caloric intake or distribution of meals. Adherence to the nutritional intervention program was assessed using a 7-day dietary record during the last week of the intervention. All foods and fluids consumed, portion sizes, how foods were prepared, and how consumption habits were distributed throughout the day were recorded. Athletes resided for 24 hours at the High-Performance Center of Barcelona, and they strictly followed the diet prescribed by medicals services from the center and the research team, with whom they were in continuous contact.

### 2.3. Exercise Energy Efficiency Stress Test

**Figure 1:**
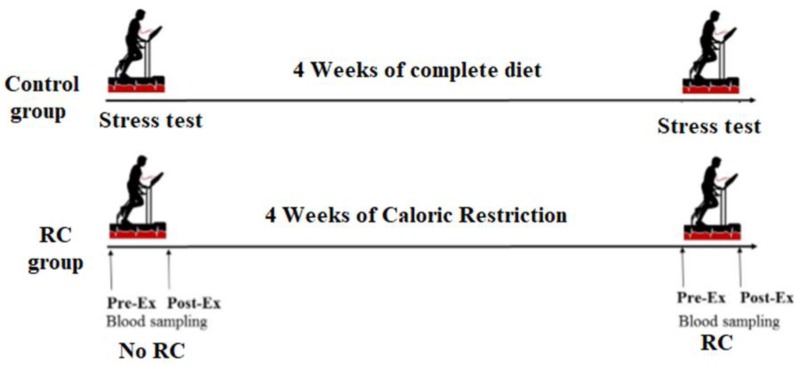
Diagram of the treatment time line. RC—restricted calorie.

Each participant completed three standardized maximal treadmill exercise tests (Figure 1). The first test was used to determine maximal running speed and maximal oxygen consumption for each athlete. The second test was used as a control of the third test, carried out after the period of calorie restriction, to determine running efficiency. Each subject performed an incremental maximal test until exhaustion on a motorized treadmill (EG2, Vitoria, Spain) to determine their maximal running speed and their maximal oxygen consumption (VO_2_max) using a computerized metabolic cart (Master Screen CPX, Erich Jaeger, Wurzburg, Germany). A fixed treadmill rise (3%) was maintained throughout the test. The treadmill running rate was initially set at 6 km/h and increased by 0.5 km/h each minute until maximum sustainable effort (muscle fatigue or stabilization/decline in VO_2_max) [28,29]. The running rate corresponding to 50%, 60%, and 70% of their VO_2_max and corresponding to the upper anaerobic threshold (if this was not under the 70% of maximum) was calculated by linear interpolation of data from the maximal exercise test. Each participant performed a maximal exercise test on the treadmill after overnight fasting at the beginning and at the end of the nutritional intervention. The athletes spent five consecutive minutes at 50%, 60%, and 70% of their VO_2_max running rate, and at the upper anaerobic threshold running rate until exhaustion. Continuous determination of O_2_ consumed (mL/min), CO_2_ expired (mL/min), and expiratory volume (L/min) was performed using a computerized metabolic cart (Master Screen CPX, Erich Jaeger, Wurzburg, Germany) and the respiratory quotient, which enables the determination of the individual contribution of fatty acids in the aerobic metabolism, was calculated [30].

### 2.4. Experimental Procedure

Four venous blood samples were obtained from the antecubital vein of each subject (pre- and post-exercise) at the beginning and at the end of the study with suitable vacutainers containing EDTA (ethylenediaminetetraacetic acid) as anticoagulant. Venous blood was collected in overnight fasted conditions (pre-exercise), and 2 h after finishing the exercise test (post-exercise) as this is coincident with an increment in circulating immune cells, with changes in antioxidant enzymes activities and in markers of oxidative damage [31]. Plasma was obtained after centrifugation (900× *g*, 30 min, 4 °C) of the blood.

### 2.5. NEFA Determinations

Plasma NEFAs were determined by a column chromatographic method followed by derivatization with reagent Meth-Prep™ II (GRACE) and subsequent gas chromatography as previously described [23,32]. The gas chromatograph was an Agilent 5890 model (Agilent Technologies, Santa Clara, CA, USA) with a flame ionization detector (FID), and the column was a Supelcowax^®^ 10 Capillary GC column, 30 m × 0.53 mm, d_f_ = 0.50 µm.

### 2.6. Enzymatic Determinations

Catalase (CAT) activity was measured in plasma by the spectrophotometric method of Aebi [33]. Superoxide dismutase (SOD) activity was measured in plasma by an adaptation of the method of McCord and Fridovich [34]. All activities were determined with a Shimadzu UV-2100 spectrophotometer at 37 °C.

### 2.7. Malonyldialdehyde Determination

Malonyldialdehyde (MDA), a marker of lipid peroxidation, was analyzed in plasma using a colorimetric assay kit (Calbiochem). Briefly, samples and standards were placed in Eppendorf tubes containing n-methyl-2-phenylindole (10.3 mM) in acetonitrile:methanol (3:1, *v*/*v*). 12 N HCl was added and the samples were incubated for 1 h at 45 °C. Absorbance was measured at 586 nm. This method is specific for MDA determination.

### 2.8. Assay of Nitrotyrosine and Protein Carbonyls

Plasma protein carbonyl derivates (10 µg of protein) and nitrotyrosine (150 µg) were determined by immunological methods using the OxiSelect™ Protein Carbonyl Immunoblot Kit (Cell Biolabs, INC) and OxiSelect™ Nitrotyrosine Immunoblot Kit (Cell Biolabs, INC) following the manufacturer’s instructions. Total protein concentrations were measured by the Bradford method [35]. Samples were transferred to a nitrocellulose membrane by the dot blot method. Image analysis was performed using Quantity One-1D analysis software (Bio-Rad Laboratories, Hercules, CA, USA).

### 2.9. Nitrite and Nitrate Determination

Plasma samples were centrifuged (15,000× *g*, 30 min, 4 °C) in 10 K filters (Amicon® Ultra; Millipore, MA, USA) to remove proteins. Supernatants were recovered and used to measure nitrite and nitrate concentration by detecting liberated NO in the gas-phase chemiluminescence reaction with ozone, using an NO analyzer (NOA 280i; Sievers, GE Power and Water, Boulder, CO, USA).

### 2.10. PBMC RNA Extraction and Real-Time PCR Assay

mRNA expression was determined by multiplex real-time PCR based on incorporation of a fluorescent reporter dye and using human 18S rRNA as reference. For this purpose, total RNA was isolated from PBMCs by Tripure extraction following manufacturer instructions (Roche Diagnostics, Germany). RNA (1 µg) from each sample was reverse transcribed using 50 U of Expand Reverse Transcriptase (Roche Diagnostics, Germany) and 20 pmol oligo (dT) for 60 min at 37 °C in a 10 µL final volume, according to manufacturer instructions. The resulting cDNA (3 µL) was amplified using the LightCycler FastStart DNA Master PLUS SYBR Green I kit (Roche Diagnostics, Germany). Amplification was performed at 55 °C and 45 cycles. The relative quantification was performed by standard calculations considering 2^(ΔΔCt)^. Gene expression were normalized to the invariant control 18S rRNA. mRNA levels at the beginning of the study were arbitrarily referred to as 1. The primers and conditions used are listed in Table 2.

### 2.11. Statistical Analysis

Statistical analysis was carried out using the Statistical Package for Social Sciences (SPSS v.21.0 for Windows). Results are expressed as mean ± standard error of the mean (SEM). The Shapiro–Wilk test was applied to verify the normality and homogeneity of variance. The statistical significance of data normally distributed was assessed by two-way analysis of variance (ANOVA) and Student’s t-test for unpaired data to determine differences between different groups, and *p* < 0.05 was considered statistically significant. Two-way ANOVA analyzed the statistical factors exercise (E) and calorie restriction (CR). Not normally distributed data was analyzed by the Friedman and Kendal test, and when significant differences between groups were found, these were identified by the Wilcoxon test.

## 3. Results

The calorie restriction intervention produced an energy intake reduction of approximately 33% with respect to energy intake in the initial condition, although the contribution of carbohydrate, protein, and fat to energy intake was maintained at the same level as the beginning (Table 1). The control group diet was isocaloric during the study period and it also maintained the initial contribution of carbohydrate, protein, and fat as the calorie restriction group. The calorie restriction intervention significantly decreased the total weight of participants by about 5.9%, corresponding to 4.6 kg, while control diet had no effects on athletes’ total weight (Table 3). The speed reached in tests performed at the beginning and at the end of the intervention was the same in control and in calorie restriction groups. On the other hand, calorie restriction significantly decreased the expiratory volume (L/min), VO_2_ (mL/min), VCO_2_ (mL/min), and the respiratory quotient during the exercise test performed at 50%, 60%, and 70% of maximal speed and at threshold, but it did not affect VO_2_ (mL/kg·min) when corrected by body weight, except for the test performed at 70% of maximal speed. The control group maintained the initial values of expiratory volume (L/min), VO_2_ (mL/min), VCO_2_ (mL/min), and respiratory quotient during the exercise tests performed at 50%, 60%, and 70% of maximal speed and at threshold (Table 3).

**Figure 2:**
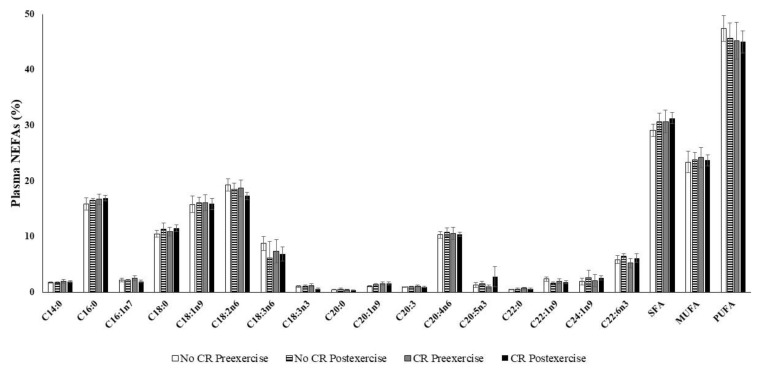
Content of non-esterified fatty acids in plasma, 20:3 was the 20:3n6 and 20:3n3 mixture. Values are represented as mean ± SEM. Statistical analysis: two-way ANOVA, *p* < 0.05. CR: Calorie restriction.

Neither calorie restriction nor exercise affected any individual fatty acid percentage or saturated fatty acid (SFA), monounsaturated fatty acid (MUFA), or polyunsaturated fatty acid (PUFA) fraction (Figure 2). The NEFA composition remained similar in the four plasma samples. Moreover, total plasma NEFAs were not significantly influenced by calorie restriction or exercise: initial pre-exercise 830 ± 58 µM, initial post-exercise 855 ± 60 µM, final calorie restriction pre-exercise 865 ± 91 µM, and final calorie restriction post-exercise 756 ± 54 µM. Plasma NEFA composition was mainly polyunsaturated (45.8% ± 1.1 %. PUFA), followed by saturated (30.4% ± 0.8 % SFAs) and monounsaturated (23.8% ± 0.8% MUFA).

Oxidative damage markers and antioxidant parameters were not significantly influenced by calorie restriction or exercise (Table 4). The markers of oxidative damage of lipids (MDA) and proteins (carbonyls) and nitrosative damage of proteins (nitrotyrosine) maintained the control values after calorie restriction and after exercise. Plasma nitrate and nitrite levels as markers of nitrate intake and nitric oxide production, respectively, also maintained the basal control levels in all the studied situations. Plasma CAT and SOD activities as markers of plasma ability to deactivate reactive oxygen species also maintained the basal initial values after calorie restriction and immediately after the exercise test.

**Figure 3:**
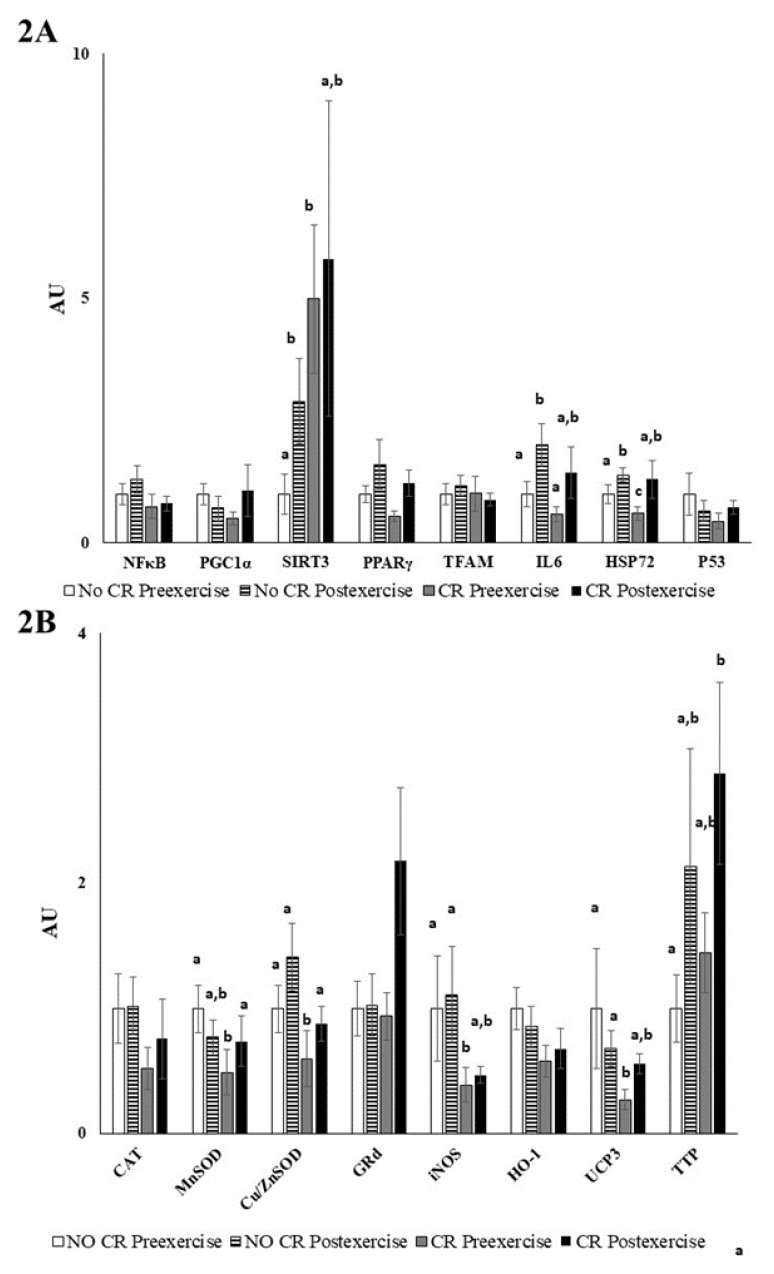
Peripheral blood mononuclear cells (PBMCs) gene expression. Values are represented as mean ± SEM. Statistical analysis: two-way ANOVA, *p* < 0.05. Different letters indicate significant differences between groups. (**A**) Inflammatory gene expression (**B**) Antioxidant gene expression

Calorie restriction significantly downregulated the gene expression of antioxidant enzymes (*MnSOD* and *Cu/Zn SOD*), enzymes related with the NO synthesis (*iNOS*), mitochondrial uncoupling proteins (*UCP3*), and repairing stress protein (heat shock protein 72, *HSP72*), as shown in Figure 3. On the other hand, calorie restriction enhanced the expression of silent information regulators (*SIRT3*) and the vitamin E carrier protein alpha-tocopherol transfer protein (*α-TTP*), which was additionally upregulated by exercise (Figure 3). In addition, neither calorie restriction nor acute exercise significantly influenced the gene expression of the antioxidant enzymes catalase, glutathione reductase and heme oxygenase (*HO-1*), or the basal expression of *NFκB, PGC1α, PPARγ, TFAM*, and *p53*. Acute exercise enhanced the gene expression of *MnSOD, UCP3*, and *α-TTP* only after the calorie restriction intervention. On the contrary, acute exercise without calorie restriction maintained the basal expression of these genes but increased the expression of the inflammatory protein *IL6* and *SIRT3*. Acute exercise in both calorie restriction and control conditions enhanced the expression of the repair stress proteins *HSP72*.

## 4. Discussion

The current study investigated the impact of a diet with a 33% calorie restriction (three alternate days a week) and physical activity (three days a week) during one month on physical performance with respect to a non-calorie restriction diet. Calorie restriction is associated with weight loss and improved health outcomes such as lower cardiometabolic disease risk and improved functional status [11]. The calorie restriction intervention reduces the total body weight and could be a nutritional intervention to manage body weight in those sports divided in weight categories. For example, wrestlers use harmful weight loss practices before competition, leading to loss of at least 2.27 kg [36]. Exercise reduces food intake by increasing the satiating efficiency of a fixed meal [37] and prevents the onset of many chronic diseases [38]. Calorie restriction enhances the energy reserve and the capacity to generate effective work when measured by an exercise energy efficiency test. Most of these benefits could be due to the weight loss of the participants, since moving a lower body weight involves lower energy requirement. However, other factors can influence the parameters of the exercise energy efficiency test, such as possible changes in the running pattern as a result of body structural changes due to body weight reduction [39] or altered fuel competition for oxidative respiration in favor of using fat as fuel [40] as we observe with the exercise energy efficiency test. Furthermore, the enhanced energy efficiency could be due to functional modifications of the mitochondrial respiratory chain [16,41,42].

Calorie restriction induces mitochondrial proliferation in rodents [43,44], and it either lowers [45] or does not affect mitochondrial oxygen consumption [43], suggesting an improvement of whole-body energy efficiency by using less oxygen and producing less mitochondrial reactive oxygen species (ROS) [16,46]. ROS are formed by single electron transfer to oxygen, generating molecules such as superoxide anion, hydrogen peroxide, and hydroxyl radical than can react with lipids, proteins, and DNA, leading to oxidative stress damage [47]. Low mitochondrial mass contributes to increase the mitochondrial workload, thus leading to higher membrane potential and increasing ROS production [43,44,47]. It has been described that calorie restriction increases mitochondrial mass and decreases proton leaks and oxidative stress damage [16,46,48]. Our results show the influence of calorie restriction in the downregulation of the basal expression of genes related with oxidative stress and inflammation. In addition, calorie restriction also modulates the response to acute exercise on the expression of these genes in PBMCs. Calorie restriction reduces the basal expression of antioxidant enzymes such as MnSOD and Cu/Zn SOD, of mitochondrial uncoupling proteins such as UCP3, of nitric oxide synthesis enzymes such as iNOS, and of repair stress proteins such as HSP72 in PBMCs. This situation might probably reflect a lower basal ROS production by PBMCs as a result of the calorie restriction [16,49]. In fact, the expression of antioxidant enzymes is enhanced by ROS, such as hydrogen peroxide, in PBMCs [49], and this response is avoided by the antioxidant vitamin C in phorbol myristate acetate (PMA)-activated neutrophils [50]. Likewise, exercise training that enhances ROS production also increases the expression of HSP72 in PBMCs of premenopausal women [51]. Our results evidence that calorie restriction in well-trained athletes modulates the response to exercise. Calorie restriction improves the antioxidant response induced by intense exercise by enhancing the expression of the mitochondrial MnSOD and cytoplasmic Cu/Zn SOD, and by enhancing the expression of the vitamin E carrier (α-TTP), in addition to enhancing the expression of HSP72. Mitochondrial MnSOD eliminates the excess of superoxide anions produced in the mitochondrial respiratory chain, while Cu/Zn SOD eliminates the excess of superoxide anions produced in cytoplasm. SODs are the antioxidant defenses that are firstly activated by acute exercise [52]. HSP72 pertains to a large family of transcriptionally regulated chaperone proteins that respond to cellular stress by assisting the response to protein damage, preventing protein aggregation, and degrading damaged proteins [53,54]. The vitamin E carrier α-TTP is expressed in the liver, brain, and in utero [55,56], but we evidence its expression also in PBMCs and its modulation by calorie restriction and exercise. α-TTP has been shown to be a determinant of vitamin E level in tissues and in circulation [57,58]. Our results suggest that calorie restriction and exercise enhance the capabilities to incorporate vitamin E into PBMCs. On the other hand, the exercise test without calorie restriction did not influence the expression of these antioxidant genes, but it enhanced the expression of the silent information regulator SIRT3, the expression of the repair stress protein HSP72 and the expression of the inflammatory IL6. SIRT3 is found in the mitochondria; it deacetylates and activates MnSOD during the presence of mitochondrial ROS being a key player in the antioxidant program [59]. Therefore, the antioxidant strategy to avoid oxidative stress and inflammation induced by acute exercise after calorie restriction intervention is quite different to the antioxidant strategy induced by acute exercise without calorie restriction. Calorie restriction promotes the elimination of ROS in the mitochondria and cytoplasm by MnSOD and Cu/Zn SOD, respectively, the incorporation of the antioxidant vitamin E by α-TTP, and the repair of damaged proteins by HSP72; whereas acute exercise without calorie restriction promotes the activation of pre-existent mitochondrial antioxidant enzymes such as MnSOD by enhancing the expression of the silent information regulator SIRT3, the induction of an inflammatory response by IL6, and the repair of damaged proteins.

In spite of the changes in gene expression, the exercise tests realized before calorie restriction and at the end of the calorie restriction intervention do not seem to compromise the plasma antioxidant/oxidant balance of the participants, as the markers of oxidative damage and the activity of antioxidant enzymes remained unchanged. Our results show the maintenance of lipid and protein oxidative damage markers and plasma antioxidant enzyme activities after the maximal exercise test performed by participants both before and after the calorie restriction period. A study performed in male non-trained adolescents with obesity showed that 4 weeks of exercise training and dietary restriction reduces body weight, increases basal antioxidant enzyme activities (SOD and glutathione peroxidase), and decreases basal protein-carbonyls [15]. We found no effects of the calorie restriction intervention for 4 weeks in well-trained athletes, but calorie restriction enhanced antioxidant defenses in obese non-trained adolescents [15]. Regular physical activity practices enhance plasma antioxidant protein levels with respect to more sedentary partakers [60]. A possible explanation to theses contradictory results is that trained sportsmen have enhanced basal antioxidant defenses as a result of intense exercise when compared to non-trained adolescents, who need an adaptation period to enhance their endogenous antioxidant defenses. Therefore, the enhancing effects of calorie restriction are not evident in trained athletes.

The calorie restriction period that reduces fatty acid availability from diet does not alter the NEFA composition of plasma, including SFAs, MUFAs, and PUFAs. Overweight and obese people, who are highly sensitive to calorie restriction, have an accelerated reduction in adiposity due to greater lipid mobilization and oxidation [61]. Acute exercise increases monounsaturated plasma NEFAs [23,62] due to stimulation of lipolysis from body fat stores in order to facilitate NEFAs availability to contracting muscle [63]. The carrying out of daily light-to-moderate physical activity is beneficial in terms of total weight and adipose reduction achieved by calorie restriction interventions [24,61]. The exercise test performed by the participants in this study did not induce changes in NEFA composition, but there were different percentages of energy contribution from glucose or fatty acids, reflected in the respiratory quotient, in the different exercise intensities and groups. Exercising sportsmen obtained more energy from fat after calorie restriction when compared to the same subjects before the intervention and to the non-calorie restriction control group. Thus, calorie restriction favors the use of fatty acids during exercise, especially at moderate intensity (50% maximal speed), while more exercise intensity involves a higher use of carbohydrates due to proximity to the anaerobic phase [30]. However, the increased use of fatty acids in the calorie restriction group is not translated into parallel changes of the plasma NEFA profile.

In conclusion, calorie restriction increases energy efficiency without modifying the NEFA profile or the plasma oxidative balance. The short-term application of this diet decreases body weight and increases physical performance. Calorie restriction also decreased the PBMCs’ basal gene expression of the antioxidant MnSOD and Cu/ZnSOD and iNOS enzymes, HSP72, and mitochondrial UCP3, whereas acute exercise induced their expression reaching values similar to those of no caloric restriction in well-trained athletes. Finally, calorie restriction attenuated the expression of the proinflammatory cytokine IL-6 after acute exercise and potentiated the expression of SIRT3 and TTP.

## Figures and Tables

**Table 1:** Diet characteristics before and after a nutritional intervention with calorie restriction.

	Control	Calorie Restriction	
		Initial	Final
Energy (Kcal)	2534 ± 639	2292 ± 137	1537 ± 84 *
Carbohydrate (% Energy)	38.8 ± 6.7	41.7 ± 2.9	41.4 ± 2.3
Protein (% Energy)	22.3 ± 7.4	18.7 ± 0.9	20.5 ± 1.0
Lipids (% Energy)	38.9 ± 15.4	39.7 ± 2.7	38.1 ± 1.7

Values are represented as mean ± SEM. Statistical analysis: Student’s test for unpaired data. (*) Significant differences between Initial and Final, *p* < 0.05.

**Table 2:** Primers and conditions used in the real-time PCRs.

Gene	Primer	Annealing Temperature
*18S*	Fw: 5′-ATGTGAAGTCACTGTGCCAG-3′	60 °C
	Rv:5′-GTGTAATCCGTCTCCACAGA-3′	
*Catalase*	Fw: 5′-TTT GGC TAC TTT GAG GTC AC-3′	60 °C
	Rv: 5′-TCC CCA TTT GCA TTA ACC AG-3′	
*Mn-SOD*	Fw: 5′CGTGCTCCCACAC ATCAATC-3′	60 °C
	Rev Rv: 5′-TGAACGTCACCG AGGAGAAG-3′	
*Cu/Zn-SOD*	Fw: 5′-TCA GGA GAC CAT TGC ATC ATT-3′	63 °C
	Rv: 5′-CGC TTT CCT GTC TTT GTA CTT TCT TC-3′	
*UCP-3*	Fw: 5′-CGT GGT GAT GTT CAT AAC CTA TG-3′	60 °C
	Rv: 5′-CGG TGA TTC CCG TAA CAT CTG-3′	
*NFκB*	Fw:5′-AAACACTGTGAGGATGGGATCTG-3′	60 °C
	Rv:5′-CGAAGCCGACCACCATGT-3′	
*PGC-1α*	Fw 5′-CAC TTA CAA GCC AAA CCA ACA ACT -3	62 °C
	Rv 5′-CAA TAG TCT TGT TCT CAA ATG GGG A-3	
*iNOS*	Fw: 5′--TCTGCAGACACGTGCGTTACT-3′	62 °C
	Rv: 5′-ATGCACAGCTGAGCATTCCA-3′	
*HSP72*	Fw: 5′-CCGGCAAGGCCAACAAGATC-3′	59 °C
	Rv: 5′-CCTCCACGGCGCTCTTCATG-3′	
*IL-6*	Fw: 5′-TACATCCTCGACGGCATCTC-3′	63 °C
	Rv: 5′-ACTCATCTGCACAGCTCTGG-3′	
*GRd*	Fw: 5′-TCA CGC AGT TAC CAA AAG GAA A-3′	64 °C
	Rev: 5′-CAC ACC CAA GTC CCC TGC ATA T-3′	
*HO-1*	Fw: 5′-CCA GCG GGC CAG CAA CAA AGT GC-3′	60 °C
	Rev: 5′-AAG CCT TCA GTG CCC ACG GTA AGG-3′	
*SIRT-3*	Fw: 5′-GAG CTT CTG GGC TGG ACA GA-3′	65 °C
	Rev: 5′-TGG GAT GTG GAT GTC TCC TAT G-3′	
*TFAM*	Fw: 5′-TCGCTCTTCCTCTGCCTAAC-3′	60 °C
	Rev: 5′-CAAGAGATGAAAACCACCTC-3′	
*PPARγ*	Fw: 5′ CCATTCTGGCCCACCAAC-3′	64 °C
	Rev: 5′-AATGCGAGTGGTCTTCCATCA-5′	
*p53*	Fw: 5′ AAGTCTGTACTTGCACG-3′	62 °C
	Rev: 5′CTGGAGTCTTCCAGTGTG-3′	
*α-TTP*	Fw: 5′-CACCCCGAAATAACACCTTC-3′	62 °C
	Rev: 5′TCGCTCTTCCTCTGCCTAAC-3′	

GRd—glutathione reductase; HO-1—heme oxygenase 1; HSP72—heat shock protein 72; iNOS—inducible nitric oxide synthase; HSP72—heat shock protein 72; IL-6—interleukin 6; NFκB—nuclear factor kappa B; PGC-1α—Peroxisome proliferator-activated receptor gamma coactivator 1-alpha; PPARγ— Peroxisome proliferator-activated receptor gamma; SIRT3—silent information regulator 3; SOD—superoxide dismutase; TFAM—mitochondrial transcription factor A; α-TTP—alpha-tocopherol transfer protein; UCP-3—Uncoupling protein 3.

**Table 3:** Performance parameters of the participants in the exercise energy efficiency tests.

Resting Condition	Control		Calorie Restriction	
	Initial	Final	Initial	Final
Weight (kg)	85.5 ± 14.3	85.8 ± 14.7	81.0 ± 1.9	76.2 ± 1.9 *
VO_2_ (mL/min)	412.1 ± 124.1	489.9 ± 152.2	408 ± 18	405 ± 20
VO_2_ (mL/min/kg)	4.9 ±1.6	5.8 ± 1.8	5.0 ± 0.2	5.3 ± 0.2
Expiratory volume (L/min)	15.1 ± 3.1	16.5 ± 4.3	12.6 ± 0.5	12.0 ± 0.5
Maximal exercise test				
Speed (km/h)	12.3 ± 1.4	12.5 ± 1.3	14.6 ± 0.4	-
VO_2_ (mL/min)	3896.8 ± 402.8	3846.8 ± 420.4	3832 ± 141	-
VO_2_ (mL/min/kg)	46.1 ± 4.8	45.5 ± 5.1	47.5 ± 1.7	-
50% maximal speed				
Speed (km/h)	6.1 ± 0.7	6.3 ± 0.7	7.7 ± 0.3	7.7 ± 0.3
VO_2_ (mL/kg.min)	27.8 ± 3.2	26.4 ± 3.7	29.5 ± 1.4	28.0 ± 1.4
Expiratory volume (L/min)	65.9 ± 8.6	63.5 ± 5.9	53.0 ± 2.2	46.0 ± 2.0 *
VO_2_ (mL/min)	2351.4 ± 239.9	2227.3 ± 189.8	2366 ± 93	2115 ± 96 *
VCO_2_ (mL/min)	2268.8 ± 265.7	2185.9 ± 164.8	2126 ± 99	1797 ± 103 *
Respiratory quotient	0.96 ± 0.04	0.98 ± 0.05	0.900 ± 0.081	0.850 ± 0.110 *
60% maximal speed				
Speed (km/h)	7.4 ± 0.9	7.5 ± 0.8	9.1 ± 0.3	9.1 ± 0.3
VO_2_ (mL/kg.min)	32.2 ± 4.1	31.9 ± 3.4	34.3± 1.4	32.9 ± 1.3
Expiratory volume (L/min)	77.4 ± 9.3	81.4 ± 11.3	65.5 ± 2.7	57.7 ± 2.1 *
VO_2_ (mL/min)	2726.7 ± 349.5	2701.5 ± 256.2	2755 ± 95	2484 ± 81 *
VCO_2_ (mL/min)	2652.1 ± 321.4	2708.3 ± 244.1	2616 ± 105	2273 ± 106*
Respiratory quotient	0.97 ± 0.04	1.003 ± 0.04	0.950 ± 0.071	0.915 ± 0.104
70% maximal speed				
Speed (km/h)	8.6 ± 1	8.8 ± 0.9	10.4 ± 0.3	10.4 ± 0.3
VO_2_ (mL/kg.min)	37.2 ± 4	36.5 ± 4.0	38.8 ± 1.6	37.1 ± 1.6 *
Expiratory volume (L/min)	97.3 ± 13.1	98.7 ± 18.3	80.0 ± 4.0	67.0 ± 2.2 *
VO_2_ (mL/min)	3154.8 ± 346.8	3083.3 ± 314.3	3117 ± 109	2804 ± 101 *
VCO_2_ (mL/min)	3201.2 ± 364.5	3185.2 ± 326.3	3067 ± 134	2617 ± 116 *
Respiratory quotient	1.02 ± 0.04	1.03 ± 0.04	0.984 ± 0.080	0.933 ± 0.241 *
Threshold				
Speed (km/h)	10.1 ± 1.3	10.5 ± 1.5	11.7 ± 0.3	11.7 ± 0.3
VO_2_ (mL/kg.min)	41.6 ± 4.5	41.2 ± 4.8	42.6 ± 1.6	40.8 ± 1.1
Expiratory volume (L/min)	123.5 ± 19.1	126.7 ± 20.4	92.6 ± 3.2	81.6 ± 3.0 *
VO_2_ (mL/min)	3530.6 ± 418.5	3471.9 ± 366.2	3428 ± 100	3112 ± 74 *
VCO_2_ (mL/min)	3830.3 ± 498.5	3838.5 ± 532.1	3427 ± 110	2959 ± 77 *
Respiratory quotient	1.09 ± 1.4	1.1 ± 0.07	1.000 ± 0.080	0.951 ± 0.081 *

CR: calorie restriction. Values are represented as mean ± SEM. Statistical analysis: Student’s test for unpaired data. (*) Significant differences between Initial and Final. *p* < 0.05.

**Table 4:** Effects of a maximal exercise test and calorie restriction intervention on plasma oxidative damage markers and antioxidant parameters.

				ANOVA		
		No CR	CR	E	CR	E × CR
Catalase (K/L)	Pre-exercise	374 ± 186	389 ± 174	0.933	0.438	0.480
	Post-exercise	207 ± 65.6	520 ± 312			
SOD (pkatal/L)	Pre-exercise	740 ± 51	717 ± 96	0.259	0.372	0.573
	Post-exercise	700 ± 56	728 ± 63			
MDA (mM)	Pre-exercise	8.27 ± 1.29	9.98 ± 2.99	0.202	0.256	0.112
	Post-exercise	8.91 ± 4.99	8.79 ± 1.65			
Carbonyl (%)	Pre-exercise	100 ± 7	101 ± 9	0.295	0.983	0.920
	Post-exercise	111 ± 11	110 ± 7			
N-Tyr (%)	Pre-exercise	100 ± 7	109 ± 5	0.096	0.121	0.943
	Post-exercise	90.5 ± 3.1	101 ± 8			
Nitrite (nM)	Pre-exercise	167 ± 10	183 ± 12	0.325	0.206	0.449
	Post-exercise	180 ± 13	186 ± 13			
Nitrate (μM)	Pre-exercise	33.5 ± 3.4	35.4 ± 3.2	0.561	0.957	0.574
	Post-exercise	33.4 ± 2.8	31.8 ± 1.8			

CR: calorie restriction, SOD: superoxide dismutase. Values are represented as mean ± SEM. Statistical analysis: two-way ANOVA, *p* < 0.05. E—significant effect of exercise, CR—significant effect of calorie restriction, E × CR—significant interaction between factors.

## Abbreviations

CAT	catalase
NEFA	non-esterified fatty acid
SOD	superoxide dismutase
